# Molecular Cues Guiding Matrix Stiffness in Liver Fibrosis

**DOI:** 10.1155/2016/2646212

**Published:** 2016-10-09

**Authors:** Takaoki Saneyasu, Riaz Akhtar, Takao Sakai

**Affiliations:** ^1^MRC Centre for Drug Safety Science, Department of Molecular and Clinical Pharmacology, Institute of Translational Medicine, University of Liverpool, Liverpool L69 3GE, UK; ^2^Centre for Materials and Structures, School of Engineering, University of Liverpool, Liverpool L69 3GE, UK

## Abstract

Tissue and matrix stiffness affect cell properties during morphogenesis, cell growth, differentiation, and migration and are altered in the tissue remodeling following injury and the pathological progression. However, detailed molecular mechanisms underlying alterations of stiffness* in vivo *are still poorly understood. Recent engineering technologies have developed powerful techniques to characterize the mechanical properties of cell and matrix at nanoscale levels. Extracellular matrix (ECM) influences mechanical tension and activation of pathogenic signaling during the development of chronic fibrotic diseases. In this short review, we will focus on the present knowledge of the mechanisms of how ECM stiffness is regulated during the development of liver fibrosis and the molecules involved in ECM stiffness as a potential therapeutic target for liver fibrosis.

## 1. Introduction

Each tissue/organ has an optimum stiffness level. The tissue/organ stiffness changes in response to biochemical and physical stimuli during the development or due to pathological conditions such as chronic fibrotic disease and cancer progression [1, 2].

Atomic force microscopy (AFM) allows surface topography of tissues to be imaged with a nanometer spatial resolution using a sharp tip attached to a cantilever. In addition to surface imaging, AFM enables the measurement of mechanical data from tip/sample interaction [3]. AFM is widely used in measuring tissue/organ stiffness at a nanoscale level and cell mechanics. The elastic modulus is typically reported using such AFM data [1, 4]. A number of studies using AFM have assessed tissue/organ stiffness and have revealed that the elastic modulus of soft tissues such as liver, lung, and skin is approximately one-fifth of the level of muscle tissues [1, 4].

Many studies suggest that ECM stiffness affects biological properties of cells and tissues. For example, naive mesenchymal stem cells cultured on soft matrices that mimic brain exhibit neurogenic phenotype, whereas those cultured on stiffer matrices that mimic muscle exhibit myogenic phenotype, suggesting that matrix elasticity governs lineage and phenotypes of stem cells [5]. NIH3T3 fibroblasts on substrates with a rigidity gradient can generate stronger traction forces and spread to a larger size on stiff substrates than on soft substrates [6]. Elevating matrix stiffness increases cell growth and disrupts epithelial morphogenesis by enhancing integrin clustering, extracellular signal-regulated kinase (ERK) activation, and Rho-generated contractility [7]. A recent insightful study shows that collagen cross-linking, which elevates tissue stiffness, leads to cancer progression by enhancing ECM receptor integrin signaling [8]. Therefore, analysis of matrix/tissue stiffness provides us with new insights in understanding the pathological mechanisms of tumor and fibrotic diseases.

Collagen is the most abundant component of scaffolding ECM in tissue/organ stroma [9, 10] and essential for macromolecular structure and organizations in the ECM. Indeed, collagen-mediated ECM networks have an effect on biological properties such as matrix/tissue stiffness and tissue/organ structure. Type I collagen is a member of fibril-forming collagen and the major molecule of collagen fibrils (more than 90%) in bone, tendon, ligament, and skin and also all major organs such as heart, kidney, liver, lung, and spleen in vertebrates [10]. Type I collagen is synthesized as procollagen and then forms fibrils after enzyme-mediated removal of both N- and C-terminus propeptides [10]. Covalent cross-linking occurs among intra- and intermolecular chain of collagens [11], which results in the stabilization and enhanced mechanical properties in extracellular collagen [12, 13]. We have recently discovered that there are at least two independent mechanisms of type I collagen fibrillogenesis in response to adult tissue/organ injury: ECM glycoprotein fibronectin-mediated and transforming growth factor- (TGF-) *β*/type V collagen-mediated [14].

TGF-*β* plays a pivotal role as a profibrogenic master cytokine in promoting differentiation of tissue-resident fibroblasts into myofibroblasts and upregulation of ECM production, including fibronectin and collagen [15, 16]. TGF-*β* is secreted into ECM as a biologically inactive (latent) complex with TGF-*β* latency-associated protein and latent TGF-*β*-binding protein- (LTBP-) 1. In response to injury, latent TGF-*β* is converted to an active form to bind its receptor. Indeed, TGF-*β* bioavailability is increased in chronic fibrotic diseases, whereas inhibition of latent TGF-*β* activation prevents the progression of fibrosis [17–20], implying that local activation of latent TGF-*β* is a critical step in the control of TGF-*β* activity. Importantly, local TGF-*β* bioavailability is negatively regulated by fibronectin following adult tissue/organ damage [14, 21].

Liver is responsible for the metabolism, synthesis, storage, and redistribution of nutrients, and it has a central role in homeostasis. Liver injury can be induced by chronic infection with hepatotropic viruses (mainly hepatitis B and C viruses) and autoimmune injury as well as by metabolic and toxic/drug-induced causes, with chronic alcohol consumption being predominant in western countries. Interestingly, the adult liver has a very high regenerative capacity. Adult liver can completely recover within weeks even after 70% resection of the total liver (partial hepatectomy) [22]. However, if liver injury persists, liver regeneration fails and this results in the excessive accumulation of collagenous ECM (mainly type I collagen, termed “liver fibrosis”). Thus, liver fibrosis is the common outcome in all chronic liver diseases. Liver fibrosis has great clinical importance because it is reversible in the early stages, before disruption of the normal liver architecture and the eventual impairment of liver function [23]. Liver cirrhosis, the end-stage irreversible consequence of liver fibrosis, causes significant morbidity and mortality and is characterized by the formation of regenerative nodules of parenchyma surrounded and separated by fibrotic septa. 170 million patients worldwide are affected by chronic liver disease, 25–30% of whom will develop significant fibrosis and eventually cirrhosis. Eventually, many patients suffer from progressive liver cirrhosis and are required to get liver transplants. Currently, there are no biomarkers that can be used to identify patients who might benefit from a specific therapy; also there are no biomarkers that can reliably predict the progression to liver fibrosis and the development of cirrhosis [24, 25].

In response to liver damage, myofibroblasts such as activated hepatic stellate cells (HSCs) play a central role in ECM remodeling [23]. In quiescent conditions, HSCs are located in the subendothelial space and store vitamin A droplets [69]. Quiescent HSCs express makers that are characteristic of adipocytes (PPAR*γ*, SREBP-1c, and leptin) [23]. Following liver injury, HSCs, like tissue-resident fibroblasts in other organs, transdifferentiate into proliferative myofibroblasts, expressing high levels of myogenic markers (alpha smooth muscle actin [*α*-SMA], c-myb, and MEF-2) and acquiring contractile, proinflammatory, and fibrogenic properties [23]. Activated HSCs proliferate and migrate to the sites for tissue repair, secreting large amounts of ECM and regulating ECM degradation [23]. Surprisingly, in advanced stages of liver fibrosis, fibrotic livers accumulate approximately up to 6 times more ECM compared to normal livers, including collagens, fibronectin, and laminin [23]. A recent* in vitro* observation has revealed that primary rat HSCs cultured for 7 days on soft substrates appear morphologically quiescent, whereas HSCs cultured on stiffer substrates exhibit typical features of myofibroblast (increased spreading and *α*-SMA expression), suggesting that alteration of liver matrix stiffness drives the pathological progression of fibrosis [70]. However, it remains to be elucidated how ECM stiffness is regulated following liver injury and how activated HSCs contribute to ECM stiffness during the development of liver fibrosis. In this short review, we will focus on the present knowledge of the regulatory mechanisms of matrix stiffness in chronic liver fibrosis.

## 2. Molecules Regulating Extracellular Matrix Stiffness

As described above, collagen cross-linking enhances the ECM stiffness [11]. Accumulating observations have identified molecules regulating protein cross-linking and ECM stiffness (Table 1). The molecules regulating ECM stiffness have relevance to chronic diseases including tissue fibrosis, neurodegenerative, autoimmune disease, and cancer [27, 71].

### 2.1. Lysyl Oxidase Family

The lysyl oxidase (LOX) family contains at least five members: LOX-like 1 (LOXL1), LOXL2, LOXL3, and LOXL4, in addition to LOX [12, 26]. They belong to an amine oxidase family and copper-dependently catalyze the posttranslational oxidation of peptidyl lysine to the peptidyl aldehyde, *α*-aminoadipic-*δ*-semialdehyde [12]. This chemical modification is known to be induced by profibrogenic cytokine TGF-*β*, enables the covalent cross-linking in fibrillar collagens and elastins, and thus results in the insolubilization and stabilization of ECM proteins [8, 72].

LOX is secreted as inactive proenzyme (proLOX) and then extracellularly cleaved to active enzyme by C-proteinase [12, 26].* In vitro* study raises the possibility that the proteolytic activation of proLOX occurs on the cell surface in a complex with cellular form of fibronectin [73]. LOX binds to cellular fibronectin at higher binding affinity (Kd = 2.5 nM) as well as type I collagen (Kd = 5.2 nM) and tropoelastin (Kd = 1.9 nM), although it is unlikely that cellular fibronectin acts as a substrate of LOX. LOX colocalizes well with cellular fibronectin in both cultured fibroblasts* in vitro* and normal human tissues* in vivo. *Interestingly, fibronectin-null embryonic fibroblasts show significant reduction of the proteolytic processing of proLOX [73]. These findings strongly suggest that fibronectin matrix regulates ECM stiffness via LOX activation.

LOX knockout mouse shows perinatal death caused by developmental abnormalities in various tissues such as diaphragm, heart, lung, skin, and vascular tissues [74–76], demonstrating that LOX is essential for normal embryonic development. In both human fibrotic diseases and animal models, elevated expression and activity of LOX family members are often observed [35, 77]. Significantly increased LOX activity is observed in sera of patients with hepatic diseases such as chronic hepatitis, fibrosis, and cirrhosis [78, 79], implying the potential of LOX family as a biomarker for liver fibrosis. LOX inhibitor *β*-aminopropionitrile (BAPN: small molecule) decreases TGF-*β*1-induced collagen fibril stiffness* in vitro* and also organ stiffness following injury [21, 80]. The treatment of BAPN with mice in carbon tetrachloride- (CCl_4_-) induced liver fibrosis facilitates fibrosis reversal after CCl_4_ withdrawal, supporting the concept of pharmacologic targeting of LOX pathway to inhibit liver fibrosis and promote its resolution [34]. LOXL2-specific inhibitory antibody reduces the extent of collagen cross-linking mediated by pSmad2/3 signaling (canonical TGF-*β*1 signaling) in mouse models of chemically induced fibrosis in the liver and lung [35] and indeed anti-LOXL2 antibody (GS-6624) in the process of clinical trials [81].

### 2.2. Transglutaminase Family

The transglutaminase- (TG-) mediated, covalent cross-linking of proteins is an essential step in tissue remodeling after injury. This process provides tissues with extra rigidity and resistance against proteolytic degradation. TGs are widely distributed calcium-dependent enzymes and catalyze covalent cross-linking between *γ*-carboxy-amine group of a glutamine residue and the *ε*-amino group of a lysine residue, resulting in a *ε*-(*γ*-glutamyl)lysine isopeptide bond [27]. Several studies indicate the involvement of TGs in human diseases such as neurodegenerative disorders, autoimmune diseases, cancer, and tissue/organ fibrosis [27]. The nine members of this family have been identified: TG1 (keratinocyte TG), TG2 (tissue TG), TG3 (epidermal TG), TG4 (prostate TG), TG5 (TGX), TG6 (TGY), TG7 (TGZ), factor XIII, and band 4.2 [82].

TG2 (tissue TG) is the most abundantly expressed member of the TGs [82]. Unlike other members, TG2 is a multifunctional protein and numerous substrates of TG2 have been identified [27, 82]. Many ECM glycoproteins (collagen, fibronectin, fibrinogen, vitronectin, laminin, and LTBP-1) are known to be the substrates of TG2. An* in vitro* study using Swiss 3T3 fibroblasts suggests the contribution of TG2 to the deposition of latent TGF-*β* complex into ECM: LTBP-1 is codistributed with extracellular TG2 and fibronectin, and increased TG2 expression elevates the deposition of LTBP-1 in the matrix along with the increase of deoxycholate-insoluble fibronectin, whereas the competitive amine substrate reduces the LTBP-1 deposition in the matrix [83]. Recent studies reveal that TG2 has not only enzymatic (cross-linking of ECM proteins) but also nonenzymatic functions [71]. The cell surface TG2 noncovalently associates with soluble fibronectin and integrin (*β*1, *β*3, and *β*5), resulting in promoting fibronectin deposition into ECM and forming stable ternary complexes with both fibronectin and integrins [71]. The association of TG2 with integrins potentially triggers outside-in signaling. Cell surface TG2 increases RhoA activity by integrin clustering and downregulation of Src-p190RhoGAP inhibitory pathway, enhancing formation of focal adhesion and actin stress fibers [71, 84]. It is therefore likely that TG2 affects ECM/tissue properties via regulating ECM cross-linking and cell-ECM interactions.

Upregulation of TG mRNA and protein levels is observed in human and murine liver fibrosis progression [36, 85]. However, TG2-knockout mice show a comparable extent and pattern of liver fibrosis compared to wild-type controls in CCl_4_- and thioacetamide-induced chronic liver injury [36]. Furthermore, reversal after CCl_4_-induced liver fibrosis is not accelerated in TG2-knockout mice. It is therefore likely that TG2 does not have a major contribution to hepatic fibrogenesis or stabilization of the collagen matrix and that TG2-independent collagen cross-linking (e.g., LOX family) could be represented as an important therapeutic target for liver fibrosis [36].

Factor XIII (FXIII) plays a central role in forming a stable fibrin meshwork by cross-linking of fibrin during blood clotting [86]. A number of studies have revealed that ECM proteins such as fibronectin, collagen (type I, II, III, and V), and vitronectin are also substrates of FXIII. For example, fibronectin is cross-linked to fibrin *α* chain by FXIII and this cross-linking produces denser clots [86]. Although fibronectin does not affect clot rigidity at physiological concentrations [86], fibronectin-fibrin cross-linking is required for fibroblast adhesion and spreading on fibronectin. However, the molecular mechanisms underlying impaired wound healing in patients lacking FXIII are still largely unknown [87, 88]. Moreover, the functional requirement for FXIII-mediated cross-linked provisional matrix between fibrin and fibronectin in adult tissue remodeling remains to be defined. We have demonstrated in an FXIII subunit A deficient murine model of acute liver injury that the lack of FXIII subunit A does not interfere with collagen reconstruction and resolution after liver injury. Furthermore, FXIIIA deficiency has caused significantly increased hepatocyte apoptosis and a delay in hepatocyte regeneration after injury, which are accompanied by a high induction of p53 expression. These findings strongly suggest a novel function of FXIII where the FXIII-mediated covalently cross-linked matrix could promote survival signals for hepatocytes in adult tissue remodeling [33].

### 2.3. A Disintegrin and Metalloproteinase with Thrombospondin Type I Motif 2 (ADAMTS2)

ADAMTS (a disintegrin and metalloproteinase with thrombospondin motif) enzymes are extracellular proteases and belong to the metzincin protease superfamily [89]. They are subgrouped on the basis of their substrates: the aggrecanases or proteoglycanases (ADAMTS1, ADAMTS4, ADAMTS5, ADAMTS8, ADAMTS9, ADAMTS15, and ADAMTS20), the procollagen N-propeptidases (ADAMTS2, ADAMTS3, and ADAMTS14), the cartilage oligomeric matrix protein-cleaving enzymes (ADAMTS7 and ADAMTS12), the von Willebrand factor proteinase (ADAMTS13), and a group of orphan enzymes (ADAMTS6, ADAMTS10, ADAMTS16, ADAMTS17, ADAMTS18, and ADAMTS19) [89]. A very recent* in vitro* study shows that ADMTS2, ADAMTS3, and ADAMTS14 cleave LTBP-1 and TGF-*β* RIII (*β*-glycan) and that ADAMTS2 silencing inhibits TGF-*β*1- or TGF-*β*2-induced expression of connective tissue growth factor and *α*-SMA in human dermal fibroblasts [90]. ADAMTS2-deficient mice show reduced hepatic fibrosis in chronic liver injury induced by CCl_4_, whereas a single CCl_4_ injection causes a similar acute liver injury in knockout and wild-type mice [37]. These findings suggest that ADAMTS2 promotes fibrosis via activation of TGF-*β* signaling and that ADAMTS2 might be a novel therapeutic target for liver fibrosis. However, it is unclear whether ADAMTS2 level is elevated in patients with liver fibrosis and whether ADAMTS inhibitors ameliorate fibrosis progression and/or accelerate the regression in animal models. Further studies remain to be elucidated to clarify the contribution of ADAMTS to ECM stiffening and progression/regression of liver fibrosis.

### 2.4. Small Leucine-Rich Proteoglycans/Protein Family

Small leucine-rich proteoglycans/protein (SLRP) family consists of five classes (I–V) and the canonical class is classes I, II, and, III including decorin, biglycan, lumican, and fibromodulin [91]. Almost all SLRPs bind collagen fibrils through their leucine-rich repeat domain. Lines of evidence show that SLRPs contribute significantly to the diameter and/or alter structure of collagen fibrils [91–93]. A dynamic modulus in biglycan-null tendons is significantly increased compared to wild-type tendons [94]. The elasticity of collagen fiber networks in cultured decorin-siRNA-transfected mouse NIH3T3 fibroblasts declines during the incubation period, whereas it is unchanged in untransfected cells [95]. It is therefore likely that SLRPs could regulate the mechanical strength of ECM.

## 3. Characterization of Liver Mechanics* In Vitro* and* In Vivo*

The role of mechanical factors in myofibroblastic activation and fibrosis has been recognized for many years [80, 96]. Hence, appropriate techniques are needed to accurately characterize the mechanical changes associated with liver fibrosis. Studies on liver mechanics have been limited due to numerous factors including small sample sizes, variable methodologies, and tissue storage methods. However, it is widely reported that liver is a viscoelastic tissue and that stiffness increases with increasing fibrosis. A range of techniques have been applied to characterize liver mechanics both* in vivo* and* in vitro*. For example, magnetic resonance elastography (MRE) has long been used for noninvasive assessment of liver fibrosis [97] and new developments of MRE allow three-dimensional spin-echo echo planar imaging [98].

For* in vitro* analysis, oscillatory rheometry which provides the complex shear modulus of liver tissue is typically used for characterization of liver stiffness and data obtained with this technique has been found to correlate with* in vivo* MRE measurements [99]. Mechanical tissue characterization with rheometry involves the analysis of the complex shear modulus. Very recently, it is shown that the shear storage (*G*′), loss (*G*′′), and apparent Young's moduli increase markedly with progressive fibrosis in rat livers [100]. They suggest that both cells and the ECM contribute significantly to the mechanical properties of the tissue and that these are driven by cell-cell and cell-ECM contacts. Whilst such approaches provide fundamental mechanical property information on biopsy samples of liver and can be used to develop a constitutive model to understand behavior [100], the information yielded for the samples is at the macroscopic/gross level. Similar to other soft tissues, in the liver, the key components of the ECM, which are altered with fibrosis, are organized at length scales which are not discriminated with conventional mechanical testing techniques [3]. Techniques such as AFM allow the mechanical properties to be probed at the nanoscale and hence open up exciting new areas of research into how the specific components of the tissue microstructure influence its mechanical behavior [3]. AFM was originally developed as a topographic imaging technique but is a highly versatile technique where the contrast in AFM images is related to the tip/sample interaction and hence the elastic properties of both tip and substrate [3]. For mechanical property measurements, AFM is typically used in force spectroscopy mode where the mechanical properties of a sample are determined with a high resolution but without correlated surface topography. A recent review by Maver et al. provides an overview of various AFM modes and its use for biomedical applications [101]. The challenges and limitations of AFM-based quantitative mechanical analysis have been reviewed elsewhere [3].

AFM has been used widely to study cell elasticity [102, 103] but to date there are few studies which have determined changes in the properties of native liver tissue with AFM. Recent applications of AFM methods to tissue highlight the utility of such AFM experiments. Zhao et al. [104] used AFM to determine the elastic modulus of biopsy samples obtained from a large cohort of patients in an investigation on the relationship between matrix stiffness and hepatocellular carcinoma. They found a positive correlation between ECM mechanical stiffness and integrin *β*1 expression, suggesting that integrin *β*1 expression is regulated by the mechanical stiffness of the ECM. Desai et al. have conducted a detailed AFM study on mice liver lobules from normal and fibrotic livers. They demonstrated that normal liver matrix stiffness was around 150 Pa but increased to 1–6 kPa in areas near fibrillar collagen deposition in fibrotic livers [105]. We have investigated whether ECM glycoprotein fibronectin could be a suitable target for ameliorating fibrosis during advanced stages of chronic liver injury, particularly focusing on the molecular mechanisms responsible for matrix stiffness [21]. We have discovered in liver fibrogenesis induced by CCl_4_ that fibronectin-null livers have exhibited constitutively elevated local TGF-*β* activity and lysyl oxidase expressions, induced more myofibroblast phenotypes, accumulated highly disorganized/diffuse collagenous ECM networks, and consequently have led to more extensive liver cirrhosis. Importantly, these phenotypes in fibronectin-null livers are accompanied by significantly elevated liver matrix stiffness, as determined by AFM, and deteriorated hepatic functions. The novel aspect of our AFM experiments is that we have simultaneously imaged the ultrastructure of the tissue and colocated the mechanical properties, using a novel AFM mechanical mapping method [106]. We have found that there is approximately a 55% increase in the elastic modulus of fibronectin-null livers compared to controls* in vivo* (5,128 ± 553.6 MPa in mutant versus 3,313 ± 835.2 MPa in control (*n* = 9); *P* < 0.05; measured at ambient conditions) [21]. Further* in vitro* mechanical integrity analysis reveals that TGF-*β*1- (2 pM-) induced collagen fibril stiffness in fibronectin-null hepatic myofibroblasts (activated HSCs) is found to be significantly higher compared to control (parental) cells. Furthermore, the treatment of fibronectin-null HSCs with 10 *μ*g/mL plasma fibronectin has recovered collagen fibril stiffness to parental cell levels* in vitro* [21]. Thus, taken together, these findings indicate that elevated TGF-*β* bioavailability in fibronectin-null livers induces more active myofibroblasts and sustains their activated phenotypes. As a consequence, these myofibroblasts develop more accumulated collagenous ECMs during advanced chronic liver damage, which thereby results in the significant deterioration of net hepatic function. We propose that there are functional links between fibronectin-mediated control of TGF-*β* bioavailability and collagen fibril stiffness.

## 4. Perspectives

Fibrosis is characterized by ECM remodeling and stiffening. Accumulating studies using animal models suggest that the molecules involved in ECM remodeling and stiffening have potential as a therapeutic target for liver fibrosis (Table 2) [107]. Several antifibrotic drugs for fibrosis, including liver fibrosis, are in the process of clinical trials [23, 81, 108]. To date, the therapeutic concept for liver fibrosis has been etiology-driven to ameliorate and eliminate the causative agents of chronic liver disease [81]. More recently, the biochemical changes affecting liver fibrosis irreversibility have become the focus, that is, direct approaches targeting specific ECMs and the extent of matrix stiffness cross-linking. For example, intravenous injection of nanoparticle loaded with procollagen *α*1(I) siRNA ameliorates progression and accelerates regression of hepatic fibrosis in mice without detectable side effects [44], suggesting that inhibition of* de novo* collagen synthesis could be a concept in the development of therapeutic agents for chronic hepatic fibrosis. Tissue-resident fibroblasts transdifferentiate into myofibroblasts in response to injury and are responsible for ECM production and remodeling. There are at least two independent mechanisms in type I collagen network organization (fibronectin- and TGF-*β*/type V collagen-mediated) in response to adult tissue/organ damage [14]. It remains to be elucidated how each mechanism contributes to matrix stiffness during the development of tissue/organ fibrosis. It also remains an unsolved question how ECM stiffness changes during the resolution of liver fibrosis and whether ECM stiffness affects the resolution process. Recent growing evidence has showed that activated HSCs are reverted to quiescent-like state both* in vitro* [109–111] and* in vivo* [112, 113], raising the possibility that lowering matrix stiffness initiates the resolution of liver fibrosis. The treatment of LOX inhibitor BAPN decreases collagen stability during liver fibrosis progression and facilitates fibrosis reversal after CCl_4_-induced advanced liver fibrosis [34]. The monoclonal antibody to LOXL2 has already been in the process of clinical trials [81]. These findings suggest that the decrease of matrix stiffness could also be a treatment strategy for hepatic fibrosis regression.

As described in this review, each of the recent studies suggests that AFM could be a powerful characterization tool to understand mechanistic changes associated with liver disease. Thus, interest in such nanoscale measurements of liver matrix stiffness during the development and progression of liver fibrosis are likely to increase in future work, particularly with the development of new imaging modalities and AFM hardware that improve the quality of data obtained for biological tissues.

## Figures and Tables

**Table 1:** Extracellular matrix stiffness-regulating molecules.

Molecules	Biological functions	References
LOX	Catalyzing cross-linking of collagen and elastin	[12, 26]
LOX-like 1–4	Catalyzing cross-linking of collagen and elastin	[12, 26]
Tissue transglutaminase	Catalyzing cross-linking of ECM proteins	[27]
Fibronectin	Decreasing LOX family expression	[21]
TGF-*β*	Increasing collagen, LOX family expression	[15, 26]
PDGF	Increasing LOX expression	[28, 29]
IL-1*β*	Increasing LOX expression	[30]
TNF-*α*	Decreasing LOX expression (1–5 ng/mL)	[31]
	Increasing LOX expression (10–30 ng/mL)	
Prostaglandin E	Decreasing LOX expression	[30]
IFN-*γ*	Decreasing LOX expression	[32]

ECM, extracellular matrix; IFN-*γ*, interferon-*γ*; IL-1*β*, interleukin-1*β*; LOX, lysyl oxidase; MMP, matrix metalloproteinase; PDGF, platelet-derived growth factor; TGF-*β*, transforming growth factor-*β*; TNF-*α*, tumor necrosis factor-*α*.

**Table 2:** Molecular targets for hepatic injury/fibrosis in animal models.

Targets	Models	Agents to induce injury/fibrosis	Administrated drug	Effects on injury/fibrosis	References
*Cross-linking factors*					
Factor XIII subunit A	Knockout mouse	CCl_4_		No effect	[33]
LOX	Wild-type mouse	CCl_4_	Small molecule inhibitor (BAPN)	Decreasing	[34]
LOX-like 2	Wild-type mouse	CCl_4_	Specific antibody	Decreasing	[35]
Tissue transglutaminase	Knockout mouse	CCl_4_		No effect	[36]
*Proteases*					
ADAMTS2	Knockout mouse	CCl_4_		Decreasing	[37]
MMP-12	Knockout mouse	Bile duct ligation		Decreasing	[38]
MMP-13	Knockout mouse	Bile duct ligation		Decreasing	[39]
Osteopontin	Knockout mouse	CCl_4_		No effect	[40]
	Overexpression mouse	CCl_4_		Increasing	[40]
Tissue-type plasminogen activator	Knockout mouse	CCl_4_		Increasing	[41]
*ECM component*					
Fibronectin	Wild-type mouse	CCl_4_, DMN	Peptide	Decreasing	[42]
	Knockout mouse	CCl_4_		Increasing	[21]
Periostin	Knockout mouse	CCl_4_		Decreasing	[43]
Procollagen *α*1(I)	Wild-type mouse	CCl_4_	siRNA	Decreasing	[44]
Thrombospondin-1	Knockout mouse	Resection		Decreasing	[45]
*Cytokines/their receptors*					
Angiotensin II type 1A receptor	Knockout mouse	CCl_4_		Decreasing	[46]
Angiotensin II type 2	Knockout mouse	CCl_4_		Increasing	[47]
Endothelin-A	Wild-type rat	Bile duct occlusion	Small molecule antagonist (LU135252)	Decreasing	[48]
TGF-*β*	Wild-type rat	Bile duct ligation	Soluble TGF-*β* receptor type II	Decreasing	[49]
	Wild-type rat	CCl_4_	BMP-7	Decreasing	[50]
	Wild-type mouse	CCl_4_	BMP-7	Decreasing	[50]
TGF-*β* type II receptor	Knockout mouse	CCl_4_		Decreasing	[51]
*Signal transduction/transcription factors*					
FXR	Wild-type rat	Porcine serum, bile duct ligation	Small molecule agonist (6-ECDCA)	Decreasing	[52]
JNK1	Knockout mouse	CCl_4_, bile duct ligation		Decreasing	[53]
MRTF-A	Knockout mouse	CCl_4_		Decreasing	[54]
PPAR*α*	Wild-type mouse	Methionine choline-deficient diet, thioacetamide	Endogenous ligand (oleoylethanolamide)	Decreasing	[55]
PPAR*γ*	Wild-type rat	CCl_4_	Small molecule agonist (pioglitazone)	Decreasing	[56]
	Wild-type mouse	CCl_4_	Small molecule agonist (pioglitazone)	No effect	[57]
	Knockout mouse	CCl_4_		Increasing	[58]
Smad3	Wild-type mouse	CCl_4_	Thyroid hormone	Decreasing	[59]
	Knockout mouse	Dimethylnitrosamine		Decreasing	[60]
*Others*					
Cannabinoid receptor CB1	Wild-type mouse	CCl_4_	Small molecule antagonist (SR141716A)	Decreasing	[61]
	Knockout mouse	CCl_4_, thioacetamide, bile duct ligation		Decreasing	[61]
Cannabinoid receptor CB2	Knockout mouse	CCl_4_		Increasing	[62]
Integrin *α*v*β*6	Wild-type rat	Bile duct ligation	Small molecule antagonist (EMD527040)	Decreasing	[63]
	Wild-type mouse	Bile duct ligation	Antibody	Decreasing	[64]
	Knockout mouse	Bile duct ligation		Decreasing	[64]
Interleukin-17 receptor	Knockout mouse	CCl_4_		Decreasing	[65]
NOX1	Wild-type mouse	CCl_4_	Small molecule inhibitor (GKT137831)	Decreasing	[66]
	Knockout mouse	CCl_4_		Decreasing	[67]
NOX4	Wild-type mouse	CCl_4_	Small molecule inhibitor (GKT137831)	Decreasing	[66]
	Knockout mouse	CCl_4_		Decreasing	[67]
Plasminogen activator inhibitor	Knockout mouse	Bile duct ligation		Decreasing	[68]

6-ECDCA, 6-*α*-ethyl-chenodeoxycholic acid; ADAMTS2, A disintegrin and metalloproteinase with thrombospondin type I motif 2; BAPN, *β*-aminopropionitrile; BMP-1, bone morphogenic protein-1; CCl_4_, carbon tetrachloride; DDC, 3,5-diethoxycarbonyl-1,4-dihydrocollidine; DMN, dimethylnitrosamine; FXR, farnesoid X-activated receptor; LOX, lysyl oxidase; MMP, matrix metalloproteinase; MRTF-A, myocardin related transcription factor A; NOX, nicotinamide adenine dinucleotide phosphate oxidase; PPAR, peroxisome proliferator-activated receptor; TGF-*β*, transforming growth factor-*β*.

## Abbreviations

6-ECDCA: 6-*α*-Ethyl-chenodeoxycholic acid
*α*-SMA: *α*-Smooth muscle actin
ADMTS: A disintegrin and metalloproteinase with thrombospondin type I motif
AFM: Atomic force microscopy
BAPN: *β*-Aminopropionitrile
BMP-1: Bone morphogenic protein-1
CCl_4_: Carbon tetrachloride
DDC: 3,5-Diethoxycarbonyl-1,4-dihydrocollidine
DMN: Dimethylnitrosamine
ECM: Extracellular matrix
ERK: Extracellular signal-regulated kinase
FXIII: Factor XIII
FXR: Farnesoid X-activated receptor
HSC: Hepatic stellate cell
IFN-*γ*: Interferon-*γ*
IL-1*β*: Interleukin-1*β*
LOX: Lysyl oxidase
LOXL: Lysyl oxidase-like
LTBP: Latent TGF-*β*-binding protein
MRE: Magnetic resonance elastography
MMP: Matrix metalloproteinase
MRTF-A: Myocardin related transcription factor A
NOX: Nicotinamide adenine dinucleotide phosphate oxidase
PPAR: Peroxisome proliferator-activated receptor
PDGF: Platelet-derived growth factor
PI3K: Phosphoinositide 3-kinase
SLRPs: Small leucine-rich proteoglycans/proteins
TGF-*β*: Transforming growth factor-*β*
TG: Transglutaminase
TNF: Tumor necrosis factor.
